# ${\text {Aut}}({\mathbb {F}}_5)$ has property (*T*)

**DOI:** 10.1007/s00208-019-01874-9

**Published:** 2019-08-08

**Authors:** Marek Kaluba, Piotr W. Nowak, Narutaka Ozawa

**Affiliations:** 1 Adam Mickiewicz University, Poznan, Poland; 2 Technische Universität, Berlin, Germany; 3 Institute of Mathematics of the Polish Academy of Sciences, Warsaw, Poland; 4 Research Institute for Mathematical Sciences, Kyoto, Japan

## Abstract

We give a constructive, computer-assisted proof that ${\text {Aut}}({\mathbb {F}}_5)$, the automorphism group of the free group on 5 generators, has Kazhdan’s property (*T*).

Kazhdan’s property (*T*) is a powerful rigidity property of groups with many applications. Few groups are known to satisfy the property and the main classes of examples are: lattices in higher rank Lie groups, whose (rigid) algebraic structure allows to deduce various properties, including property (*T*); groups acting on complexes whose links satisfy a certain spectral condition, including groups acting on buildings; and certain hyperbolic groups, such as lattices in ${\text {Sp}}(n,1)$ and some random hyperbolic groups in the Gromov density model.

The main purpose of this article is to prove property (*T*) for a new group and give an estimate of its Kazhdan constant. Let ${\text {SAut}}({\mathbb {F}}_{5})$ denote the special automorphism group of the free group on 5 generators, with *S* being its standard generating set consisting of transvections.

## Theorem 1

The group ${\text {SAut}}({\mathbb {F}}_{5})$ has property (*T*) with Kazhdan constant

$$\begin{aligned} \kappa ({\text {SAut}}({\mathbb {F}}_{5}), S) > 0.18. \end{aligned}$$

As part of the proof we provide explicit elements $\xi _i$ in the group ring of ${\text {SAut}}({\mathbb {F}}_{5})$ and $\lambda > 0$ such that

$$\begin{aligned} \Delta ^2-\lambda \Delta \approx \sum _{i=1}^n \xi _i^*\xi _i, \end{aligned}$$

up to a small, controlled error. The $\xi _i$s are obtained via semidefinite optimization. Then we show that there exists a mathematically exact solution (for a slightly smaller $\lambda$) in close proximity of the approximate one.

This method of proving property (*T*) was forseen by the third author in [29] and used effectively by Netzer and Thom [26], Fujiwara and Kabaya [12] and the first two authors [18] to reprove property (*T*) and give new estimates for Kazhdan constants for the groups ${\text {SL}}_n({\mathbb {Z}})$, $n=3,4,5$. We describe in detail the theoretical aspects, the algorithm which is used to produce the solution, as well as the certification procedure that allows to obtain, out of this approximate solution, a mathematically correct conclusion about the existence of an exact one. In particular we describe the modifications to the algorithm of [18] which made a search for $\xi _i$s in the context of ${\text {SAut}}({\mathbb {F}}_{5})$ and the certification of the result technically possible.

As an immediate consequence of Theorem 1 we obtain

## Corollary 2

The groups ${\text {Aut}}({\mathbb {F}}_5)$ and ${\text {Out}}({\mathbb {F}}_5)$ have property (*T*).

Questions whether any of the groups above has property (*T*) is discussed in many places, e.g. [6, Question 7], [22, 5, p 345], [4, p 4], [9, p 63], to name a few. Applications to the product replacement algorithm were discussed in [22].

The group ${\text {Aut}}({\mathbb {F}}_n)$ is known not to have property (*T*) for $n=2$ and $n=3$ see [24] as well as [4, 15]. It was suspected nevertheless that ${\text {Aut}}({\mathbb {F}}_n)$ might have property (*T*) for *n* sufficiently large, with all $n\geqslant 4$ being open. In the case of $n=4$ our approach—both the one in [18] and the symmetrized version presented below – did not give a positive answer, in the sense that we were not able to obtain a sufficiently approximate solution on the ball of radius 2. There are three possible reasons for this behavior. One possibility is that ${\text {Aut}}({\mathbb {F}}_4)$ does not have property (*T*) (as somehow anticipated by [4]). Another is that ${\text {Aut}}({\mathbb {F}}_4)$ has property (*T*), however no $\xi _i$ as above, supported on the ball of radius 2 exist. Search for $\xi _i$s supported on the ball of radius 3 is already too expensive (in terms of memory and computation time) to be handled by our implementation. Finally, the third possibility is that again ${\text {Aut}}({\mathbb {F}}_4)$ has property (*T*) which is wittnessed on the ball of radius 2, but the spectral gap is so small, that the certification process does not yield a positive answer.

It is an interesting question whether the fact that ${\text {Aut}}({\mathbb {F}}_5)$ has property (*T*) is sufficient to deduce property (*T*) for ${\text {Aut}}({\mathbb {F}}_n)$ for all $n\geqslant 5$, similarly as in the case of lattices in higher rank Lie groups. In Sect. 6 we show that ${\text {Aut}}({\mathbb {F}}_{n+1})$ has a large subgroup with property (*T*), if ${\text {Aut}}({\mathbb {F}}_n)$ has (*T*). However, all attempts to prove property (*T*) for ${\text {Aut}}({\mathbb {F}}_n)$, $n\geqslant 6$ using property (*T*) for ${\text {Aut}}({\mathbb {F}}_5)$ seem to break down at the currently open Question 12 in [6].

It was proved in [14] that for $n\geqslant 3$ the group ${\text {Out}}({\mathbb {F}}_n)$ is residually finite alternating and it is a consequence of Theorem 1 that the corresponding family of alternating quotients of ${\text {Out}}({\mathbb {F}}_5)$ can be turned into a family of expanders. This was proved earlier in greater generality by Kassabov [20], however in our case the generating set is explicit and the same in all of these finite groups, in the sense that it is the image of the generating set of ${\text {Out}}({\mathbb {F}}_5)$. In particular, this gives an alternative and independent of [20] negative answer to question [21, Question 10.3.2].

Theorem 1 also allows us to give an answer to a question of Popa on the existence of certain crossed product von Neumann algebras with property (T), see Remark 12.

*Note:* The techniques developed here became later crucial in [17] while proving property (*T*) for ${\text {Aut}}(F_n)$ for all $n\geqslant 6$. However, the case $n=5$ is not accessible via the argument in [17] and the only existing proof of the case $n=5$ is in the current paper.

## Property (*T*), real algebraic geometry and semidefinite programming

Recall that a group *G* generated by a finite set *S* has property (*T*) if there exists $\kappa >0$ such that

$$\begin{aligned} \sup _{s\in S} \Vert \pi _s v-v\Vert \geqslant \kappa \Vert v\Vert , \end{aligned}$$

for every unitary representation $\pi$ of *G* with no non-zero invariant vectors. The supremum of all such $\kappa$’s that satisfy the condition above is called the Kazhdan constant of *G* with respect to the generating set *S* and is denoted $\kappa (G,S)$. For an excellent overview of property (*T*), its many descriptions and applications see [2].

Let *G* be a discrete group generated by a finite set $S=S^{-1}$. Given a ring *R* we consider the associated group ring *RG*, that consists of finitely supported functions $\xi :G\rightarrow R$. We will use the notation $\xi =\sum _{g\in G} \xi _g g$, where $\xi _g\in R$ for each $g\in G$, to denote the elements of the group ring *RG*. The product in *RG* is then defined by the convolution $(\xi \eta )_g=\sum _{h\in G} \xi _{h}\eta _{h^{-1}g}$. Recall that the augmentation ideal *IG* is the kernel of the augmentation map $\omega :RG\rightarrow R$, $\xi \mapsto \sum _{g\in G} \xi _g$. The group ring *RG* is equipped with an involution $^*:RG\rightarrow RG$, induced by the inversion map on *G* and defined explicitly as $(\xi ^*)_g=\xi _{g^{-1}}$ for any $\xi \in R G$.

The unnormalized Laplacian is an element of the real group ring ${\mathbb {R}}G$ defined as

$$\begin{aligned} \Delta = \vert S\vert - \sum _{s\in S} s. \end{aligned}$$

In [29] the following characterization of property (*T*) for discrete groups was proved.

### Theorem 3

A finitely generated group *G* has Kazhdan’s property (*T*) if and only if there exist a positive number $\lambda >0$ and a finite family $\{\xi _1, \ldots , \xi _n\}$ of elements of the real group ring ${\mathbb {R}}G$ such that

1 $$\begin{aligned} \Delta ^2-\lambda \Delta = \sum _{i=1}^n \xi _i^*\xi _i. \end{aligned}$$

The characterization above can be used to prove property (*T*) for a particular group *G* by providing an explicit solution of Eq. (1). In particular, the fact that the right hand side is a finite sum of (hermitian) squares of finitely supported functions allows us to try to obtain the $\xi _i$’s using semidefinite programming.

Let $E\subseteq G$ be a finite subset and let $B_r(e,S)$ denote the ball (centered at *e*) of radius *r* in the word-length metric on *G* induced by the generating set *S*. Although some of the following considerations are true for arbitrary *E*, we use $E = S\cup S^2=B_2(e,S)$ (or $E=B_3(e,S)$) in practice. Fix ${\mathbf {x}}$, an ordered basis of the finite dimensional subspace $\langle E\rangle _{\mathbb {R}} \subseteq {\mathbb {R}}G$. Then Eq. (1) has a solution in $\langle E\rangle _{\mathbb {R}}$ if and only if there exists a semi-positive definite matrix *P* such that

$$\begin{aligned} \Delta ^2-\lambda \Delta = {\mathbf {x}}^* P {\mathbf {x}}^T. \end{aligned}$$

Indeed, by positive semidefiniteness *P* can be written as $P=QQ^T$ and then

$$\begin{aligned} {\mathbf {x}}^* P {\mathbf {x}}^T = ({\mathbf {x}}Q)^*({\mathbf {x}}Q)^T = \sum \xi _i^*\xi _i, \end{aligned}$$

where $\xi _i={\mathbf {x}}q_i$ for $q_i$ the *i*-th column of *Q*. In what follows we will drop the distinction and use $\xi _i$ for both a column of a matrix and the corresponding group algebra element without mentioning the basis ${\mathbf {x}}$.

Let ${\mathbb {M}}_E$ denote the set of real matrices with columns and rows indexed by *E*. For $t\in G$ define a matrix $\delta _t \in {\mathbb {M}}_E$ by setting

$$\begin{aligned} \big (\delta _t\big )_{x,y}=\left\{ \begin{array}{ll} 1 &{} \quad \text { if } \,\, x^{-1}y=t\\ 0 &{} \quad \text { otherwise}. \end{array}\right. \end{aligned}$$

Equivalently, this is the element $t\in G$ viewed as an endomorphism of $\langle E \rangle _{{\mathbb {R}}}$ defined by left-regular representation of *G* on ${\mathbb {R}} G$. For matrices $A,B\in {\mathbb {M}}_E$ define

$$\begin{aligned} \langle A,B\rangle = \sum _{x,y\in E} A_{x,y} B_{x,y}={\text {tr}}(A^T B), \end{aligned}$$

where ${\text {tr}}(A)=\sum _{x\in E} A_{x,x}$ is the standard trace on ${\mathbb {M}}_E$. Then

$$\begin{aligned} \langle \delta _t,P\rangle =\sum _{x^{-1}y=t} P_{x,y}, \end{aligned}$$

and for every $t\in E^{-1}E$ the value $(\Delta ^2-\lambda \Delta )_t$ at *t* can be expressed as

$$\begin{aligned} (\Delta ^2-\lambda \Delta )_t= \langle \delta _t, P\rangle . \end{aligned}$$

This reduces the problem of existence of a solution to Eq. (1) to the following semidefinite optimization problem in standard form (we use $P \succcurlyeq 0$ to denote the constraint of *P* being positive semidefinite).

OP $$\begin{aligned} \begin{aligned} \text {minimize} \quad&-\lambda \\ \text {subject to} \quad&P\succcurlyeq 0,\quad P\in {\mathbb {M}}_E,\\&\langle \delta _t, P\rangle = (\Delta ^2 - \lambda \Delta )_t\quad \text {for all } t\in E^{-1}E. \end{aligned} \end{aligned}$$

There are *solvers* specialized in solving such problems numerically. Once a solution $(P, \lambda _0)$ is obtained numerically (up to specified precision), the next step is to *certify* its correctness. This is of utmost importance, as the numerical solution by itself does not provide mathematical certainty that Eq. (1) indeed has a solution in ${\mathbb {R}} G$. The solution $(P, \lambda _0)$ gives only $\Delta ^2 - \lambda _0 \Delta \approx {\mathbf {x}}P{\mathbf {x}}^T$, an approximate equality. E.g. “positive semidefinite” matrix *P* returned by the solver may have negative eigenvalues which are very close to 0 (i.e. up to the requested precision), or the linear constraints defined by $\Delta ^2 - \lambda \Delta$ might be slightly violated. Moreover, even though some solvers claim to certify the solution, it is done in floating point arithmetic, which provides no mathematical certainty, see [27]. Our certification process turns the approximate solution into a proof of the existence of an exact solution, at the cost of decreasing $\lambda _0$.

The process consists of finding (the real part of) the square root *Q* of *P* (i.e. $QQ^T \approx P$), and projecting the obtained matrix *Q* onto the augmentation ideal, simply by subtracting the mean value of the *i*-th column of *Q* from each entry in that column. This procedure, e.g. performed in rational (multiprecision) arithmetic, provides an explicit matrix ${\overline{Q}}$, whose columns correspond to elements of the augmentation ideal supported on *E*. In our case this is done in interval arithmetic as described in detail later. This process introduces additional error into each element of ${\mathbf {x}}^*{\overline{Q}}\left( {\mathbf {x}}{\overline{Q}}\right) ^T = \sum _i \xi _i^*\xi _i$, which in general depends on the accuracy obtained by the chosen numerical solver. However (and most importantly) after the projection, Lemma 4 below allows to dominate $r = \Delta ^2 - \lambda _0\Delta - \sum _i \xi _i^*\xi _i$, the remainder of the solution. Note that *r* gathers both the inaccuracy of the solver and the error introduced by the projection.

For $\xi \in {\mathbb {R}}G$ let $\Vert \xi \Vert _1=\sum _{g\in G}\vert \xi _g\vert$ be the norm of $\xi$ in $\ell _1(G)$. We write $a \geqslant b$ for elements *a*, *b* of a group ring to denote that $a-b$ enjoys a decomposition into sum of (hermitian) squares. The following lemma allows to estimate the magnitude of errors involved in our computations and makes certification possible. For a more thorough treatment of order units see [29], as well as [16, 31] for a more general context.

### Lemma 4

[26, 29] $\Delta$ is an order unit for the augmentation ideal *IG*, i.e. for all self-adjoint elements $r\in IG$ there exists $R_0$ such that for all $R\geqslant R_0$ we have

2 $$\begin{aligned} r+R\Delta \geqslant 0 \end{aligned}$$

that is, $r+R\Delta$ allows a sum of squares decomposition.

Moreover, if *r* is supported on $B_{2^{m}}$ then

$$\begin{aligned} R_0 \leqslant 2^{2m-1}\Vert r\Vert _1. \end{aligned}$$

(If *S* contains no involution, then $R_0\leqslant 2^{2m-2}\Vert r\Vert _1$ suffices).

This allows to conclude that $r +\varepsilon \Delta \geqslant 0$, for an appropriately chosen $\varepsilon >0$ and thus

$$\begin{aligned} \Delta ^2 - (\lambda _0 - \varepsilon ) \Delta = \sum _i \xi _i^*\xi _i + r + \varepsilon \Delta \geqslant 0. \end{aligned}$$

When $\varepsilon$ can be chosen sufficiently small in relation to $\lambda _0$ (so that $\lambda _0-\varepsilon >0$), we can conclude that Eq. (1) indeed has a solution in ${\mathbb {R}}G$.

### Complexity of the problem

The size of the set *E* translates directly to the computational complexity of optimization problem (OP): while each element $\xi _i$ is (by its definition) supported on *E*, Eq. (1) defines $|E^{-1}E|$ linear constraints and $|E|^2$ variables in one semidefinite constraint of size $|E|\times |E|$. It seems to be an interesting problem to understand the influence of the choice of the set *E* on the obtained bound $\lambda _0$ and numerical properties of the problem.

The optimization problem has been solved numerically in several cases in [12, 18, 26] yielding new estimates for $\lambda$. The computations were successful for $E=B_2(e,S)$ e.g. for the groups ${\text {SL}}_n({\mathbb {Z}})$ with $n=3,4,5$. Finding a numerical solution of the problem on a computer may not be feasible if the size of the set *E* is too large, and in fact this is the case for the groups ${\text {SL}}_n({\mathbb {Z}})$, $n\geqslant 6$, or the groups ${\text {SAut}}({\mathbb {F}}_{n})$ when $n\geqslant 4$. For instance, the case of ${\text {SAut}}({\mathbb {F}}_{5})$ results in 21, 538, 881 variables and 11, 154, 301 constraints, making it a prohibitively large problem. In order to remedy this and to be able to find a solution to optimization problem (OP) e.g. for ${\text {SAut}}({\mathbb {F}}_{5})$, we will use the symmetries of the set *E* to reduce the problem’s size.

## Problem symmetrization

The size and computational complexity of optimization problem (OP) can be significantly decreased by exploiting its rich symmetry derived from the group structure. Roughly speaking, we will replace solving a large problem by solving many smaller problems and patching the solutions together to obtain a solution to the original, larger problem. While there are $|E|^2$ variables in the original problem, the number of variables of the symmetrized version is $m_1^2+\cdots +m_k^2$, where $m_k$ are the dimensions of the individual component problems. Note that $\sum _i m_i \leqslant |E|$ i.e. the latter is much smaller than the former. Moreover using orbit constraints we will reduce the number of constraints significantly. In the case of ${\text {SAut}}({\mathbb {F}}_{5})$ the result is a problem consisting of 13,232 variables in 36 semidefinite constraints and 7229 linear constraints, which can be realistically attacked with a numerical solver. However, to be able to perform our computations we will need to give a different parametrisation of the space of possible solutions by the means of orbit reduction and the Wedderburn decomposition.

A somewhat parallel exposition of semidefinite programs size reduction using its symmetry is discussed in detail in [1, 7]. We would like to point out that a numerical approach to numerical symmetrization of optimization problems that does not use group representation theory is described in [25], which may be applicable e.g. to finitely presented groups where the symmetry is not clearly visible.

### Invariant SDP problems

Given an automorphism $\sigma$ of *G* and $\xi \in R G$, denote by $\sigma (\xi )$ the element of *RG* defined by $\sigma (\xi )_g=\xi _{\sigma (g)}$. Let $\Sigma _E$ denote the group

$$\begin{aligned} \Sigma _E:=\left\{ \sigma \in {\text {Aut}}(G): \sigma (\Delta )=\Delta \text { and } \sigma (E) =E\right\} . \end{aligned}$$

The group $\Sigma _E$ is finite, determined by its image in ${\text {Sym}}(S)$, the symmetric group on *S*. Let $\Sigma$ denote any subgroup of $\Sigma _E$. The action of $\Sigma$ on *E* induces an action of $\Sigma$ on ${\mathbb {M}}_E$ by the formula

$$\begin{aligned} (\sigma (T))_{x,y} =T_{\sigma ^{-1}(x),\sigma ^{-1}(y)}. \end{aligned}$$

The subspace of matrices invariant under this action will be denoted ${\mathbb {M}}_E^\Sigma$.

#### Lemma 5

The expression $\Delta ^2-\lambda \Delta$ is invariant under $\Sigma$.

#### Proof

The verification is straightforward:

$$\begin{aligned} (\Delta ^2 -\lambda \Delta )_{\sigma (t)}&= \sum _{g\in G} (\Delta -\lambda I)_g \Delta _{g^{-1} \sigma (t)}\\&= \sum _{g\in G} (\Delta -\lambda I)_g \Delta _{\sigma \left( \sigma ^{-1}(g^{-1})t\right) }\\&= \sum _{g\in G} (\Delta -\lambda I)_{\sigma ^{-1}(g)} \Delta _{\sigma ^{-1}(g^{-1})t}\\&=(\Delta ^2 -\lambda \Delta )_t, \end{aligned}$$

since, in particular, $\sigma ^{-1}$ is a bijection. $\square$

#### Lemma 6

We have $\delta _{\sigma (t)}=\sigma (\delta _t)$ and $\delta _{t^{-1}}=\delta _t^T$. $\square$

In [1] a semidefinite problem is said to be invariant with respect to an action of a group *G* if for every solution *P*, *gP* is also a solution for every $g\in G$.

#### Proposition 7

Optimization problem (OP) is $\Sigma$-invariant.

#### Proof

Let $P\in {\mathbb {M}}_E$ be a solution to (OP). We need to show that $\sigma (P)$ is also a solution to the same problem for every $\sigma \in \Sigma$; i.e.,

$$\begin{aligned} (\Delta ^2-\lambda \Delta )_t= \langle \delta _t, \sigma (P)\rangle , \end{aligned}$$

for every $t\in E^{-1} E$. We have

$$\begin{aligned} \langle \delta _t, \sigma (P)\rangle&= \sum _{x^{-1}y=t} P_{\sigma ^{-1}(x),\sigma ^{-1}(y)}\\&=\sum _{\sigma (x')^{-1}\sigma (y')=t} P_{x',y'}\\&=\sum _{\sigma (x'^{-1}y')=t} P_{x',y'}\\&=\langle \delta _{\sigma ^{-1}(t)}, P\rangle . \end{aligned}$$

The latter is equal to $(\Delta ^2-\lambda \Delta )_{\sigma ^{-1}(t)}=(\Delta ^2-\lambda \Delta )_t$, by Lemma 5. $\square$

In particular, convexity yields

#### Corollary 8

Let $P\in {\mathbb {M}}_E$ be a solution to problem (OP) for some $\lambda >0$. Then there exists $P\in {\mathbb {M}}_E^{\Sigma }$ that also solves (OP) for the same $\lambda >0$.

The corollary above shows that we may as well search for an invariant solution.

### Orbit symmetrization

Since $\Delta ^2-\lambda \Delta$ is $\Sigma$-invariant, it is well defined on the orbit space $E^{-1}E\big /\Sigma$. While we can decompose *E* and $E^{-1}E$ into the orbits of $\Sigma$, it is impossible to naively formulate problem (OP) in “orbit variables” $E\big /\Sigma$, as the constraint matrices $\delta _t$ are not well defined in ${\mathbb {M}}_{E/\Sigma }$. However, since the solution *P* is $\Sigma$-invariant, constraint matrices can be averaged over orbits. Let $[t]_{\Sigma } = [t]$ denote the orbit of $t\in E^{-1} E$ under the action of $\Sigma$. We define

$$\begin{aligned} \delta _{[t]} = \frac{1}{|\Sigma |}\sum _{\sigma \in \Sigma } \delta _{\sigma (t)}, \end{aligned}$$

which encodes $\left( \Delta ^2-\lambda \Delta \right) _{[t]}$, the value of $\Delta ^2-\lambda \Delta$ at (any point of) [*t*]. The orbit symmetrization of problem (OP) can then be written as:

$$\begin{aligned} \begin{aligned} \text {minimize} \quad&-\lambda \\ \text {subject to} \quad&P \succcurlyeq 0, P \in {\mathbb {M}}_E \\&\left( \Delta ^2 - \lambda \Delta \right) _{[t]} = \left\langle \delta _{[t]}, P \right\rangle \quad \text {for all}\quad [t]\in E^{-1}E/\Sigma . \end{aligned} \end{aligned}$$

The problem reduces the number of constraints in problem (OP) from $\left| E^{-1}E\right|$ to $\left| E^{-1}E\big /\Sigma \right|$. However, since *P* constitutes just one semidefinite constraint of size $|E|\times |E|$, and each constraint is expressed by $|E|\times |E|$-matrix multiplication, the problem is still numerically hard to solve efficiently.

### Block-diagonalization via Wedderburn decomposition

Recall that the orthogonal dual ${\widehat{G}}$ of a group *G* is the family of equivalence classes of irreducible orthogonal representations of *G*. We will work under the assumption that all irreducible characters of $\Sigma$, the subgroup of the group of symmetries of *E*, are real. By $n{\mathbf {1}}_{\mathbb {R}}$ we denote the *n*-fold direct sum of the trivial, 1-dimensional real representation.

Let $\varrho _E$ denote the representation of $\Sigma$ on $\ell _2(E)$ induced by the permutation action of $\Sigma$ on *E*. We can decompose (up to unitary equivalence) $\varrho _E \cong \bigoplus _{\pi \in {\widehat{\Sigma }}} m_\pi \pi ^{}$ into $\pi$-isotypical summands, where each irreducible representation $\pi$ occurs with multiplicity $m_\pi$. Since the matrix algebra $C^*(\varrho _E)$ (generated by $\varrho _E(\sigma )$ for $\sigma \in \Sigma$) is semisimple, we can find its Wedderburn decomposition: an isomorphism of $C^*(\varrho _E)$ and a direct sum of simple matrix algebras:

$$\begin{aligned} C^*(\varrho _E) \rightarrow \bigoplus _{\pi \in {\widehat{\Sigma }}} {\mathbb {M}}_{\dim \pi } \otimes m_{\pi } {\mathbf {1}}_{{\mathbb {R}}} . \end{aligned}$$

As $\Sigma$-invariant matrices in ${\mathbb {M}}_E$ coincide with $\left( C^*(\varrho _E)\right) '$, the commutant of the algebra $C^*(\varrho _E)$, we obtain

$$\begin{aligned} {\mathbb {M}}_E^\Sigma \cong \left( C^*(\varrho _E)\right) ' \rightarrow \bigoplus _{\pi \in {\widehat{\Sigma }}} \dim \pi {\mathbf {1}}_{{\mathbb {R}}} \otimes {\mathbb {M}}_{m_{\pi }} . \end{aligned}$$

We show how to explicitly realize and block-diagonalize the Wedderburn isomorphism on ${\mathbb {M}}_E^\Sigma$ by using a *minimal projection system* in ${\mathbb {R}}\Sigma$. Denote by $\chi _\pi$ the central projection in ${\mathbb {R}}\Sigma$ defined by the character of an irreducible representation $\pi$. Let $\left\{ p_\pi \right\} _{\pi \in {\widehat{\Sigma }}}$ be a minimal projection system; i.e., a set of self-adjoint, primitive idempotents in ${\mathbb {R}}\Sigma$ satisfying $p_\psi p_\pi = 0$ for $\psi \ne \pi$ and $\chi _\psi p_\pi = \delta _{\psi ,\pi }p_\pi$, where $\delta _{\psi ,\pi }$ denotes the Kronecker delta.

It follows from the definition of $p_\pi$ that the image of $\varrho _E(p_\pi )$ is a subspace of $\ell _2(E)$ of dimension $m_\pi$.

Let $U_\pi$ be the matrix realizing the orthogonal projection from $\ell _2(E)$ onto ${\text {im}} \rho _E(p_\pi )$, followed by an isometric isomorphism from ${\text {im}} \rho _E(p_\pi )$ onto ${\mathbb {R}}^{m_\pi }$. For $A\in {\mathbb {M}}_E^\Sigma$ we define

$$\begin{aligned} \Theta _{\pi } (A) = \dim \pi \cdot U_\pi A U_\pi ^T. \end{aligned}$$

Under the Wedderburn isomorphism above we have

$$\begin{aligned} A \mapsto&\bigoplus _{\pi \in {\widehat{\Sigma }}}\ \dfrac{1}{\dim \pi }\cdot \left( \dim \pi {\mathbf {1}}_{{\mathbb {R}}}\right) \otimes \Theta _\pi (A), \end{aligned}$$

and thus the map

$$\begin{aligned} A \mapsto&\bigoplus _{\pi \in {\widehat{\Sigma }}} \Theta _\pi (A) \end{aligned}$$

defines a block-diagonalizing linear isomorphism

$$\begin{aligned} \Theta = \bigoplus _{\pi } \Theta _{\pi } :{\mathbb {M}}_E^\Sigma \rightarrow&\bigoplus _{\pi }{\mathbb {M}}_{m_\pi }, \end{aligned}$$

which satisfies $${\text {tr}}(A) = \sum _{\pi \in {\widehat{\Sigma }}} {\text {tr}}(\Theta _{\pi }(A))$$.

### Symmetrization

For the purposes of numerical computation, instead of working with $\Theta$ and a basis of ${\mathbb {M}}_E^\Sigma$ it is advantageous to work with the standard bases of each of ${\mathbb {M}}_{m_\pi }$ and translate the constraints to the new basis via $\Theta$.

The following optimization problem is a counterpart of problem (OP) combining both the orbit symmetrization and the block-diagonalization reduction:

SOP $$\begin{aligned} \begin{aligned} \text {minimize}\quad&-\lambda \\ \text {subject to} \quad&\{P_\pi \succcurlyeq 0, \quad P_\pi \in {\mathbb {M}}_{m_{\pi }} \}_{\pi \in {\widehat{\Sigma }}}, \\&(\Delta ^2-\lambda \Delta )_{[t]} = \sum _{\pi \in {\widehat{\Sigma }}} \left\langle \Theta _{\pi } (\delta _{[t]}), P_{\pi }\right\rangle \text { for } [t]\in E^{-1}E\big /\Sigma . \end{aligned} \end{aligned}$$

This allows for a reduction of the number of variables in problem (OP) from $|E|^2$ to $\sum _{\pi } m_\pi ^2$ (which turns out to be drastically smaller in practice) at the cost of increasing the numerical complexity of the constraints. Moreover, as most solvers can exploit the block-diagonal structure, the sizes of matrices the solver needs to compute with are much decreased.

#### Proposition 9

Let $\left( \lambda _0, \{P_\pi \}_{\pi \in {\widehat{\Sigma }}}\right)$ be a solution to problem (SOP). Then $(\lambda _0, P)$ is a solution to problem (OP), where

$$\begin{aligned} P=\frac{1}{|\Sigma |}\sum _{\sigma \in \Sigma }\sum _{\pi \in {\widehat{\Sigma }}} \dim {\pi }\cdot \sigma \left( U_{\pi }^T P_{\pi } U_{\pi }\right) . \end{aligned}$$

#### Proof

All we need to check is that $\langle \delta _t, P\rangle = \left( \Delta ^2 - \lambda _0 \Delta \right) _t$ for all $t\in E^{-1}E$. We have

$$\begin{aligned} \langle \delta _t, P\rangle&= \left\langle \delta _t, \dfrac{1}{\vert \Sigma \vert } \sum _{\sigma \in \Sigma } \sum _{\pi \in {\widehat{\Sigma }}} \dim \pi \cdot \sigma \left( U_\pi ^T P_\pi U_\pi \right) \right\rangle \\&= \dfrac{1}{\vert \Sigma \vert } \sum _{\sigma \in \Sigma } \sum _{\pi \in {\widehat{\Sigma }}} \dim \pi \left\langle \delta _t, \sigma \left( U_\pi ^T P_\pi U_\pi \right) \right\rangle \\&= \dfrac{1}{\vert \Sigma \vert } \sum _{\sigma \in \Sigma } \sum _{\pi \in {\widehat{\Sigma }}} \dim \pi \cdot {\text {tr}}\left( \delta _{\sigma ^{-1}(t)}^T U_\pi ^T P_\pi U_\pi \right) \\&= \dfrac{1}{\vert \Sigma \vert } \sum _{\sigma \in \Sigma } \sum _{\pi \in {\widehat{\Sigma }}} {\text {tr}}\left( \dim \pi \cdot U_\pi \delta _{\sigma ^{-1}(t)}^T U_\pi ^T P_\pi \right) \\&= \sum _{\pi \in {\widehat{\Sigma }}} \left\langle \dim \pi \dfrac{1}{\vert \Sigma \vert } \sum _{\sigma \in \Sigma } U_\pi \delta _{\sigma ^{-1}(t)}U_\pi ^T, P_\pi \right\rangle \\&= \sum _{\pi \in {\widehat{\Sigma }}} \left\langle \dim \pi \cdot U_\pi \delta _{[t]} U_\pi ^T, P_\pi \right\rangle . \end{aligned}$$

Recalling that $\Theta _\pi \left( \delta _{[t]}\right) = \dim \pi \cdot U_\pi \delta _{[t]} U_\pi ^T$ and using the formulation of the costraints of problem (SOP) we continue

$$\begin{aligned} = \sum _{\pi \in {\widehat{\Sigma }}} \left\langle \Theta _{\pi }\left( \delta _{[t]}\right) , P_\pi \right\rangle = (\Delta ^2 - \lambda _0\Delta )_{[t]} = (\Delta ^2 - \lambda _0\Delta )_t. \end{aligned}$$

$$\square $$

## The group ${\text {Aut}}({\mathbb {F}}_n)$

### Presentation for ${\text {Aut}}({\mathbb {F}}_n)$

Let $n\in {\mathbb {Z}}$ be positive and consider the corresponding free group ${\mathbb {F}}_n$ on *n* generators. Denote by ${\text {Aut}}({\mathbb {F}}_n)$ the group of automorphisms of ${\mathbb {F}}_n$. The *special automorphims group*${\text {SAut}}({\mathbb {F}}_n)$ is defined as the preimage of $\{1\}$ under the map

$$\begin{aligned} {\text {det}}:{\text {Aut}}({\mathbb {F}}_n)\rightarrow \lbrace -1,+1\rbrace , \end{aligned}$$

obtained by the composition

$$\begin{aligned} {\text {Aut}}({\mathbb {F}}_n) \rightarrow {\text {Aut}}({\mathbb {Z}}^n)={\text {GL}}_n({\mathbb {Z}})\rightarrow {\mathbb {Z}}. \end{aligned}$$

The first arrow above is induced by the abelianization homomorphism ${\mathbb {F}}_n\rightarrow {\mathbb {Z}}^n$, and the second is the classical determinant. The group ${\text {SAut}}({\mathbb {F}}_n)$ is of index 2 in ${\text {Aut}}({\mathbb {F}}_n)$. Denote by ${\text {Inn}}({\mathbb {F}}_n)\leqslant {\text {Aut}}({\mathbb {F}}_n)$ the subgroup of inner automorphisms of ${\mathbb {F}}_n$. The *outer automorphism group*${\text {Out}}({\mathbb {F}}_n)$ is defined as the quotient ${\text {Out}}({\mathbb {F}}_n)={\text {Aut}}({\mathbb {F}}_n){/}{\text {Inn}}({\mathbb {F}}_n)$.

Let $\left( s_1,\ldots , s_n \right)$ be an ordered set of generators of ${\mathbb {F}}_n$ and consider the following maps of ${\mathbb {F}}_n$:

$$\begin{aligned} R^{\pm }_{i,j} (s_k)&= {\left\{ \begin{array}{ll} s_ks_j^{\pm 1} &{} \text { if } k=i,\\ s_k &{} \text { otherwise;} \end{array}\right. } \end{aligned}$$

and

$$\begin{aligned} L^{\pm }_{i,j} (s_k)&= {\left\{ \begin{array}{ll} s_j^{\pm 1} s_k &{} \text { if } k=i,\\ s_k&{} \text { otherwise.} \end{array}\right. } \end{aligned}$$

The set $S = \lbrace R^{\pm }_{i,j}, L^{\pm }_{i,j}\text { for }1\leqslant i,j \leqslant n, i\ne j \rbrace$ acts on generating *n*-tuples of ${\mathbb {F}}_n$ and consists of automorphisms of ${\mathbb {F}}_n$ which generate the group ${\text {SAut}}({\mathbb {F}}_n)$ [13, 22]. The group ${\text {Aut}}({\mathbb {F}}_n)$ is generated by automorphism in *S* together with automorphisms permutating and inverting generators of ${\mathbb {F}}_n$. For a detailed description of automorphisms in *S* (commonly known as Nielsen transformations or transvections) see [23, Section 3.2].

### Implementation of ${\text {Aut}}({\mathbb {F}}_n)$

When computing in ${\text {Aut}}({\mathbb {F}}_{n})$ one usually choses to represent elements either as words in a finite presentation, or actual functions on ${\mathbb {F}}_n$ transforming free generating sets of ${\mathbb {F}}_n$ to free generating sets. The former aproach allows the group operations to be purely mechanical operations on symbols, but the recognition of the identity element (the word problem) is a major obstruction to effective computation. The latter approach, requires storage of both the domain (a generating *n*-tuple) and the image of the domain. It provides an easier solution to the recognition of the identity problem: two automorphisms are equal if and only if their values on the standard generating set of ${\mathbb {F}}_n$ agree. However, to compute those values one needs to “equalize” the domains and compare the images of the domain under both automorphims. The final step requires only trivial cancellation.

Instead of using either of the approaches we decided to produce our own implementation, where each element carries both the structure of a word in a finite presentation, as well as functional information. Let $\Gamma _n$ be the graph with the vertex set consisting of generating *n*-tuples of ${\mathbb {F}}_n$ and an edge connecting two such tuples if one can be obtained from the other by the application of an automorphism $s\in S$ (we label the edge by *s*). We represent elements of ${\text {Aut}}({\mathbb {F}}_n)$ as paths in the graph $\Gamma _n$ which start at the standard *n*-generating tuple $(x_1 , \ldots , x_n )$. Such path is represented by a word over alphabet *S* by collecting edge labels in a natural fashion. Therefore each such path determines an automorphism of ${\mathbb {F}}_n$ which takes the tuple $(x_1 , \ldots , x_n )$ (the initial vertex of the path) to the *n*-generating tuple of the terminal vertex.

Given two automorphisms $f = f_1 \ldots f_k$, $g = g_1 \ldots g_{l}$ (written as words over alphabet *S*), to decide the equality problem it is enough to compare

$$\begin{aligned} f((x_1, \ldots , x_n))&= (x_1 ,\ldots x_n)^{f_1 \ldots f_{k}} \quad \text {and} \\ g((x_1, \ldots , x_n))&= (x_1 ,\ldots x_n)^{g_1 \ldots g_{l}}, \end{aligned}$$

where letters $f_i$ of *f* ($g_j$ of *g*) act on the tuple from the right.

Effectively, we represent ${\text {Aut}}({\mathbb {F}}_n)$ as a finitely presented group on *S* and solve the word problem in an indirect manner. Note that in this setting it is sufficient to store in each letter only minimal amount of information (namely: its type (*R*, or *L*), two indicies (*i*, *j*) and the exponent (±)), as storage of neither the reference basis nor the image is required.

### The symmetrizing group $\Sigma$

The group generated by the automorphisms permuting and inverting generators of ${\mathbb {F}}_n$ is isomorphic to the group ${\mathbb {Z}}/_2\wr S_n$ (the signed permutation group). Consider the action of ${\mathbb {Z}}/_2\wr S_n$ on ${\text {Aut}}({\mathbb {F}}_n)$ by conjugation. Clearly the action preserves the set *S* of all Nielsen transformations and hence the group ${\text {SAut}}({\mathbb {F}}_{n})$. Moreover the action preserves the word-length metric on ${\text {SAut}}({\mathbb {F}}_{n})$ induced by *S* (and thus $E=B_2(e,S)$), so we can set $\Sigma = {\mathbb {Z}}/_2\wr S_n$ in our considerations.

#### Minimal projection system for $\Sigma$

Since $\Sigma \cong {{\mathbb {Z}}_2}^n \rtimes S_n$ is a semi-direct product of $S_n$ acting in the natural fashion on the *n*-fold direct product of ${\mathbb {Z}}_2$, we may obtain a minimal projection system for $\Sigma$ from a minimal projection system of $S_n$. It is a well known fact (see e.g. [32, Section 8.2]) that irreducible representations of the semidirect product are formed in the following fashion. The dual group ${\widehat{{\mathbb {Z}}_2}}^n$ (which equals the character group in this case) decomposes into $(n+1)$ orbits of the induced action of $S_n$. Let $g_i \in {\widehat{{\mathbb {Z}}_2}}^n$ denote the character which evaluates to $-1$ on the non-trivial element in the first *i* coordinates and to 1 otherwise. Then $\left\{ g_i\right\} _{i=0}^n$ forms a complete set of orbit representatives of the action, and the stabiliser of $g_i$ is $H_i = S_i\times S_{n-i}$, embedded naturally into $S_n$ (under the convention $S_0 = S_1 = \{{\text {id}}\}$). It is now straightforward that every irreducible representation of $\Sigma$ is of the form

$$\begin{aligned} \theta _{i,\pi } = {\text {ind}}_{{{\mathbb {Z}}_2}^n\rtimes H_i}^\Sigma g_i\otimes \pi , \end{aligned}$$

where $g_i\in \widehat{{\mathbb {Z}}_2}^n$ and $\pi \in \widehat{H_i}$ are trivially extended to ${{\mathbb {Z}}_2}^n\rtimes H_i$.

A minimal projection system for ${{\mathbb {Z}}_2}^n\rtimes H_i$ can be described as

$$\begin{aligned} \left\{ \chi _i p_\pi \right\} _{\pi \in \widehat{H_i}}, \end{aligned}$$

where $\chi _i$ denotes the central projection associated to $g_i$ and $\left\{ p_\pi \right\} _{\pi \in \widehat{H_i}}$ is a minimal projection system for $H_i$. Note that irreducible representations of $H_i = S_i\times S_{n-i}$ are tensor products of irreducible representations of the factors, thus a minimal projection system for $H_i$ can be constructed from those of $S_i$ and $S_{n-i}$, by taking all possible products of minimal projections. Accordingly,

$$\begin{aligned} \{ \{\chi _i p_\pi p_\psi \}_{\pi \in \widehat{S_i}, \psi \in \widehat{S_{n-i}}}\}_{i=0}^n \end{aligned}$$

is a minimal projection system for $\Sigma$.

#### Minimal projection system for $S_n$, $n\leqslant 6$

We present a minimal projection systems for $S_n$, $n\leqslant 6$ which we used in our computations. It is well known that irreducible representations of $S_n$ correspond to integer partitions of *n*. For the trivial and the sign representations (partitions $n_1$ and $1_n$, respectively) we pick $\varepsilon = ()$. For other representations $\pi$ we fix the element $\varepsilon _\pi$ as in the table

**Table:** 

*n*	Partition	$\varepsilon _\pi$
3	$2_1 1_1$	$\frac{1}{2}\big (() + (1,2)\big )$
4	$2_1 1_2$	$\frac{1}{2}\big (() + (1,2)\big )$
	$3_1 1_1$	$\frac{1}{2}\big (() - (1,2)\big )$
	$$2_2 $$	$$\frac{1}{2}\big (() + (1,2)\big )$$
5	$$2_1 1_3 $$	$$\frac{1}{2}\big (() + \text {(}1,2\text {)}\big )$$
	$$3_1 1_2 $$	$$\frac{1}{4}\big (() + (1,4,3,2) + (1,3)(2,4) + (1,2,3,4)\big )$$
	$$2_2 1_1 $$	$$\frac{1}{3}\big (() + (1,3,2) + (1,2,3)\big )$$
	$$4_1 1_1 $$	$$\frac{1}{2}\big (() - \text {(}1,2\text {)}\big )$$
	$$3_1 2_1 $$	$$\frac{1}{3}\big (() + (1,3,2) + (1,2,3)\big )$$
6	$$2_1 1_4 $$	$$\frac{1}{2} \big (() + (1,2)\big )$$
	$$3_1 1_3 $$	$$\frac{1}{4} \big (() + (1,2) + (1,2)(3,4) + (3,4)\big )$$
	$$2_2 1_2 $$	$$\frac{1}{5} \big (() + (1,3,5,2,4) + (1,4,2,5,3) + (1,5,4,3,2) + (1,2,3,4,5)\big )$$
	$$4_1 1_2 $$	$$\frac{1}{4} \big (() - (1,2) + (1,2)(3,4) - (3,4)\big )$$
	$$3_1 2_1 1_1 $$	$$\frac{1}{12} \big (() + (1,2) + (1,2)(3,4) + (3,4) + (3,4,5) + (1,2)(3,4,5) + $$
		$$(1,2)(3,5) + (3,5) + (1,2)(4,5) + (4,5) + (3,5,4) + (1,2)(3,5,4)\big )$$
	$$5_1 1_1 $$	$$\frac{1}{2} \big (() - (1,2)\big )$$
	$$2_3 $$	$$\frac{1}{3} \big (() + (1,3,2) + (1,2,3)\big )$$
	$$4_1 2_1 $$	$$\frac{1}{5} \big (() + (1,3,5,2,4) + (1,4,2,5,3) + (1,5,4,3,2) + (1,2,3,4,5)\big )$$
	$$3_2 $$	$$\frac{1}{3} \big (() + (1,3,2) + (1,2,3)\big )$$

Recall that $\chi _\pi$ is the central projection corresponding to the irreducible (character of the) representation $\pi$. By inspecting the appropriate table of characters one can check that $\varphi _\pi ( \varepsilon _\pi ) = 1$ for the character $\varphi _\pi$ corresponding to $\pi$. Even though projections $\varepsilon _\pi$ are not necessarily mutually orthogonal, it follows from the orthogonality of characters that

$$\begin{aligned} \left\{ p_{\pi } = \chi _\pi \varepsilon _\pi \right\} _{\pi \in \widehat{S_n}} \end{aligned}$$

constitutes a minimal projection system for $S_n$.

## Description of the algorithm

Given a group *G* generated by a symmetric set *S* and a finite subgroup $\Sigma$ of automorphisms of *G* preserving *S*:

1. generate $E=B_2(e,S)$, $E^{-1}E = B_4(e,S)$ and $\Delta$ (stored in delta.jld as the coefficients vector in $E^{-1}E$);
2. compute the division table $E^{-1}\times E \rightarrow E^{-1}E$ (stored in pm.jld);
3. compute the permutation representation $\varrho _E:\Sigma \rightarrow {\mathbb {M}}_E$ (stored in preps.jld);
4. compute the Wedderburn decomposition of ${\mathbb {M}}_E$, i.e. for every $\pi \in {\widehat{\Sigma }}$ compute $U_\pi$’s (stored in U_pis.jld)
5. decompose $E^{-1}E$ into orbits of $\Sigma$ (stored in orbits.jld);
6. use $\Delta$, the orbit structure, the division table and $U_\pi$’s to construct the constraints of symmetrized optimization problem (SOP);
7. solve the symmetrized optimization problem to obtain $\big (\lambda _0, \{P_\pi \}_\pi \big )$ (stored in lambda.jld, and SDPmatrix.jld);
8. reconstruct *P* according to Proposition 9 (stored in SDPmatrix.jld);
9. certify the solution $(\lambda _0, P)$ as described in Sect. 4.3.

### Division table on *E*

To perform quickly the multiplication $\xi _i^*\xi _i$ we cache the division table $M:E^{-1}\times E \rightarrow E^{-1}E$, as a matrix $M\in {\mathbb {M}}_E$ such that $M_{g,h} = g^{-1}h$. To avoid indexing entries of *M* by group elements and storing them in *M* (due to technical reasons) we fix a non-decreasing (with the word-length) order ${\mathbf {x}}$ of elements in $E^{-1}E$, i.e. such that ${\mathbf {x}}_0 = e$, $\{{\mathbf {x}}_j :j \in \{1, \ldots , |S|\}\}=S$, and $E \subseteq E^{-1}E$ as the first |*E*|-elements. Then we can store only the (integer) indices of elements in the division table, i.e. if $g \in E$ is the *i*-th element of $E^{-1}E$, ($g = {\mathbf {x}}_i$) and $h\in E$ is the *j*-th element of $E^{-1}E$ ($h={\mathbf {x}}_j$), then $M_{i,j} = k$, where $g^{-1}h = {\mathbf {x}}_k$ is the *k*-th element of $E^{-1}E$. Thus once the full *M* has been populated, we no longer need the actual group elements to perform (twisted^1^) multiplication of elements of ${\mathbb {R}}G$ supported on *E*. In particular, given a solution $(\lambda _0, P)$ of problem (OP), division table *M* and $\Delta$ (as vector of values on ${\mathbf {x}}$), one can compute the sum of squares decomposition $\sum _i \xi _i^*\xi _i$ and compare it with $\Delta ^2 - \lambda _0 \Delta$ without the need to access group elements directly.

Note that the full division table is also needed for producing the constraint matrices.

### Symmetrization

The minimal projection system $\{p_\pi \}_{\pi \in {\widehat{\Sigma }}}$ for $\Sigma$ is computed in ${\mathbb {Q}}\Sigma$ as described in Sect. 3.3. Then coefficients are converted to floating point numbers and $\varrho _E(p_\pi )$ is evaluated, where $\varrho _E$ is the permutation representation of $\Sigma$ on *E*. Matrix representatives for $U_\pi$ are obtained from $\varrho _E(p_\pi )$ using singular value decomposition.

### Certification

The certification process starts by converting the solution of problem (SOP) to a solution $(\lambda _0, P)$ of problem (OP) using standard floating-point arithmetic. Then certification of $(\lambda _0, P)$ follows mostly the procedure described in [18, Section 2]. However, instead of passing through rational approximation of $Q = \sqrt{P}$, as described there, we use the interval arithmetic directly. We turn each entry of *Q* into an interval of radius $\varepsilon \sim 2.2\cdot 10^{-16}$ which contains the computed value. Then we shift those intervals so that the sum of each colum $q_{i}$ contains 0 (this corresponds to the projection to the augmentation ideal). Setting $\xi _i=q_i{\mathbf {x}}$, as previously, we finally compute the residual

$$\begin{aligned} r = \Delta ^2-\lambda _0\Delta - \sum \xi _i^*\xi _i. \end{aligned}$$

Note that in this approach each $\xi _i$ as well as *r* is an element of the group ring over real intervals. While by using interval arithmetic we loose track of the actual values of $\xi _i$ and *r*, we have a *mathematical guarantee* that there are $\overline{\xi _i}$ with rational coefficients, $\overline{\xi _i}\in \xi _i$ (with $\in$ on the coefficient level), such that the result ${\overline{r}}$ of a similar computation performed in rational arithmetic (with $\overline{\xi _i}$’s and $\overline{\lambda _0}$) satisfies

$$\begin{aligned} \Vert {\overline{r}}\Vert _1 \in \Vert r\Vert _1 = \left[ r_{\textit{low}}, r_{\textit{up}}\right] . \end{aligned}$$

Although less precise than rational arithmetic, the computation of $\Vert r\Vert _1$ from *Q* as described above is much faster, as it involves multiplication, addition of (machine) floating point numbers, and directed rounding. Performing similar calculations in rational arithmetic is much slower (if possible in the case of ${\text {SAut}}({\mathbb {F}}_{5})$ at all), as the numerator of a sum of near-zero rationals grows exponentially.

### Software details

Our implementation in Julia programming language [3] depends on the following packages

- AbstractAlgebra [11] package for the general framework and computations involving special linear groups;
- JuMP [8] package for formulation of the optimization problem;
- SCS [28] solver for solving semi-definite problems.

Additional software has been developed to perform the presented computations. While developed for the purpose of this paper, these are not specifically tied to it and serve the general purpose as described.

- package Groups.jl for computations in wreath products and automorphism groups of free groups;
- package GroupRings.jl for computations in group rings (with basis);
- package PropertyT.jl for computations of the spectral gap;

Finally git-repository 1712.07167 contains the specific environment to reproduce the computational results. Please consult  this notebook for an example use.

### Replication details

The ball of radius 4 in ${\text {SAut}}({\mathbb {F}}_{5})$ consists of 11,154,301 elements. Generating $B_4(e, S)$, decomposing it into 7229 orbits of ${\mathbb {Z}}_2\wr S_5$ action, computing division table on $B_2(e, S)$ and finding $U_\pi$’s for the Wedderburn decomposition takes about 3 h and requires at most $20\hbox {GB}$ of RAM.^2^ Once this has been computed, the actual optimization problem consists of 13,232 variables in 36 semi-definite blocks and 7230 constraints. The optimization phase had been running for over 800 h until the acuracy of $10^{-12}$ has been reached. Note that a much shorter running time is possible if one additionally constraints $\lambda$ from above, by say 1.0. The reconstruction of *P* (according to Proposition 9) and its certification take approximately 1.5 h in total. The replication of the results should be possible on a reasonably modern desktop computer (times reported correspond to a workstation with 4-core CPU).

The pre-computed division table, orbit decomposition, $U_\pi$’s, as well as the solution *P* used in the proof of Theorem 1 can be obtained from [19].

## Proof of Theorem 1

We are now in position to prove our main theorem. As indicated above we set $E = B_2(e,S)$ and obtained a solution of optimization problem (SOP).

### Proof of Theorem 1

Let a solution $(\lambda _0 , P)$ of optimization problem (OP) be given. Compute *Q* the real part of the square root of *P*. Construct ${\overline{Q}}$ as described in Sect. 4.3 and let $\xi _i$ be the *i*-th column of ${\overline{Q}}$. Recall that the residual is given by

$$\begin{aligned} r=\Delta ^2-\lambda _0\Delta -\sum \xi _i^*\xi _i, \end{aligned}$$

and that the $\ell _1$-norm $\Vert r\Vert _1 = [r_{\textit{low}}, r_{\textit{up}} ]$ is an interval. By Lemma 4 we have $r + 2^2 r_\textit{up} \Delta \geqslant 0$ hence

$$\begin{aligned} \Delta ^2- (\lambda _0-2^2 r_{\textit{up}} )\Delta \geqslant \sum \xi _i^*\xi _i \geqslant 0. \end{aligned}$$

This allows to conclude that the spectral gap satisfies

$$\begin{aligned} \lambda (G,S)\geqslant \lambda _0-2^2 r_{\textit{up}} . \end{aligned}$$

In the case of the provided solution for ${\text {SAut}}({\mathbb {F}}_5)$ we have

$$\begin{aligned} \lambda _0 = 1.3, \quad \text {and} \quad \Vert r \Vert _1 \subset [8.30\cdot 10^{-6}, 8.43\cdot 10^{-6}], \end{aligned}$$

which leads to certified estimate $\lambda > 1.2999$. Since the generating set consists of 80 elements this results in

$$\begin{aligned} \kappa ({\text {SAut}}({\mathbb {F}}_{5}), S) > 0.18027. \end{aligned}$$

$$\square $$

## Extrapolation of property (*T*)

In case of arithmetic groups, once we know ${\text {SL}}_n({\mathbb {Z}})$ has property (*T*) for some *n*, it is rather easy to deduce from this fact that ${\text {SL}}_m({\mathbb {Z}})$ has property (*T*) for all $m\geqslant n$, because ${\text {SL}}_m({\mathbb {Z}})$ is boundedly generated by finitely many conjugates of ${\text {SL}}_n({\mathbb {Z}})$ (see [33], particularly around Section 4.III.7).

However, in the case of ${\text {Aut}}({\mathbb {F}}_n)$ a similar approach seems to break down at the currently open Question 12 in [6]. Namely, it is not known whether a quotient *Q* of ${\text {Aut}}({\mathbb {F}}_{n+1})$ must be finite provided that ${\text {Aut}}({\mathbb {F}}_n)$ has finite image in *Q*. If a counterexample exists, property (*T*) of ${\text {Aut}}({\mathbb {F}}_n)$ cannot, obviously, tell anything about such an infinite quotient *Q*. We nevertheless make an effort to extrapolate property (*T*) of ${\text {Aut}}({\mathbb {F}}_n)$ to a larger group.

For a group *G*, a normal subgroup $H\leqslant G$ and a unitary representation $(\pi ,{\mathscr {H}})$ of *G*, let ${\mathscr {P}}_H$ denote the orthogonal projection onto the space ${\mathscr {H}}^H$ of vectors invariant under $\pi (H)$. The definition of $\kappa (G,S,\pi )$ from Sect. 1 (where *S* is a subset of *G*) can be reformulated as the supremum of $\kappa \geqslant 0$ that satisfy

$$\begin{aligned} \kappa \Vert v- {\mathscr {P}}_G v \Vert \leqslant \max _{s\in S} \Vert v - \pi _s v\Vert \end{aligned}$$

for all $v \in {\mathscr {H}}$. Recall that a group *G* has property (*T*) if and only if there is a finite (necessarily generating, see [2, Proposition 1.3.2]) subset *S* such that the corresponding Kazhdan constant $\kappa (G,S,\pi )$ is strictly positive for every $\pi$.

Let $\alpha :G\rightarrow {\text {Aut}}(H)$, $g\mapsto \alpha _g$, be an action and $T\subseteq H$ be a subset such that

$$\begin{aligned} T_G= \lbrace \alpha _g(t) :g \in G,\,t\in T \rbrace \end{aligned}$$

generates *H*. Put

$$\begin{aligned} \ell (h) = \min \lbrace k :\exists s_1,\ldots ,s_k\in T_G\cup T_G^{-1} \text{ such } \text{ that } h=s_1\cdots s_k \rbrace . \end{aligned}$$

### Proposition 10

Let $G\ltimes H$ be the semidirect product given by an action $\alpha :G\rightarrow {\text {Aut}}(H)$ and $\ell$ be as above. Assume that *T* is a finite generating subset of *H*, ${{\,\mathrm{Inn}\,}}(H)$ is a subgroup of $\alpha (G)$ and

$$\begin{aligned} L=\max \left\{ \ell (t^m) :t\in T,\, m\in {\mathbb {N}} \right\} <\infty . \end{aligned}$$

Then, for any unitary representation $(\pi ,{\mathscr {H}})$ of $G\ltimes H$, one has

$$\begin{aligned} \kappa (G\ltimes H,S\cup T,\pi ) \geqslant \dfrac{\kappa (G,S,\pi |_G)}{1+4|T|^{1/2}L}. \end{aligned}$$

In particular, if *G* has property (*T*) or $(\tau )$, then so does $G\ltimes H$.

### Proof

Let $\kappa = \kappa (G,S,\pi |_G)\leqslant 2$ denote the Kazhdan constant of *G* with respect to representation $(\pi |_G, {\mathscr {H}})$. If $\kappa = 0$ there is nothing to prove, so let us assume that $\kappa >0$. Given $v\in {\mathscr {H}}$ we can define

$$\begin{aligned} C=\max _{g\in S\cup T} \Vert v - \pi _g v\Vert . \end{aligned}$$

If $w = {\mathscr {P}}_G v$ is the orthogonal projection of *v* onto the subspace of $\pi |_G$-invariant vectors in ${\mathscr {H}}$ then $\Vert v - w \Vert \leqslant \kappa ^{-1}C$. Moreover, for every $g\in G$ and $t\in T$, one has

$$\begin{aligned} \Vert w - \pi _{\alpha _g(t)}w \Vert = \Vert w - \pi _{gtg^{-1}}w \Vert = \Vert w - \pi _t w \Vert \leqslant 4\kappa ^{-1}C. \end{aligned}$$

Thus, for every $t\in T$

$$\begin{aligned} \sup _m \big \Vert w - \pi _{t^m}w \big \Vert \leqslant 4\kappa ^{-1}LC,\\ \end{aligned}$$

which implies

$$\begin{aligned} \left\| \left( 1 - {\mathscr {P}}_{\langle t\rangle }\right) w \right\| \leqslant 4\kappa ^{-1}LC, \end{aligned}$$

for the orthogonal projection ${\mathscr {P}}_{\langle t\rangle }$ onto ${\mathscr {H}}^{\langle t \rangle }$.

Since *w* is *G*-invariant and ${{\,\mathrm{Inn}\,}}(H) \leqslant \alpha (G)$, the linear functional $\tau (a)=\langle aw,w \rangle$ is a trace on the von Neumann algebra *M* generated by $\pi (H)$. Namely, $\tau (a^*a)=\tau (aa^*)$ for any element $a \in M$. In particular, if orthogonal projections *p* and *q* are Murray–von Neumann equivalent in *M*, i.e., $p = v^*v$ and $q = vv^*$ for some $v \in M$, then one has $\tau (p) = \tau (q)$. We write $p \sim q$ to indicate that *p* and *q* are Murray–von Neumann equivalent in *M*. We note that for any orthogonal projections $p,q\in M$ one has

$$\begin{aligned} p-p\wedge q \sim p\vee q -q, \end{aligned}$$

where $p\wedge q$ (respectively, $p\vee q$) is the orthogonal projection onto ${{\,\mathrm{ran}\,}}p\cap {{\,\mathrm{ran}\,}}q$ (respectively, ${{\,\mathrm{{\overline{span}}}\,}}({{\,\mathrm{ran}\,}}p \cup {{\,\mathrm{ran}\,}}q)$). Here ${{\,\mathrm{ran}\,}}p$ denotes the range of *p* and ${{\,\mathrm{{\overline{span}}}\,}}(x)$ the closure of the linear subspace spanned by set *x*.

We have

$$\begin{aligned} \Vert (p-p\wedge q)w\Vert ^2 = \tau (p-p\wedge q) = \tau (p\vee q -q) = \Vert (p\vee q -q)w\Vert ^2, \end{aligned}$$

see [34, Proposition V.1.6] for a proof of this fact. In particular,

$$\begin{aligned} \left\| \left( 1-p\wedge q\right) w\right\| ^2 \leqslant \left\| \left( 1-p\right) w\right\| ^2+\left\| \left( p\vee q-q\right) w\right\| ^2 \leqslant \left\| \left( 1-p\right) w\right\| ^2+\left\| \left( 1-q\right) w\right\| ^2 \end{aligned}$$

(the first inequality follows from the triangle inequality and the equality above; the second is the consequence of $p\vee q - q \leqslant 1 - q$). Since ${\mathscr {P}}_{H}=\bigwedge _{t\in T}{\mathscr {P}}_{\langle t\rangle }$, it follows that

$$\begin{aligned} \big \Vert \left( 1-{\mathscr {P}}_H\right) w\big \Vert \leqslant \left( \sum _{t\in T}\left\| \left( 1-{\mathscr {P}}_{\langle t\rangle }\right) w\right\| ^2 \right) ^{1/2} \leqslant 4\kappa ^{-1}\left| T\right| ^{1/2}LC. \end{aligned}$$

Since ${\mathscr {H}}^H$ is *G*-invariant (by normality of *H*), ${\mathscr {P}}_H w$ is still *G*-invariant. Therefore,

$$\begin{aligned} \big \Vert v - {\mathscr {P}}_G v \big \Vert \leqslant \big \Vert v - {\mathscr {P}}_H w \big \Vert \leqslant \kappa ^{-1} (1+4|T|^{1/2}L)C. \end{aligned}$$

This proves the claim. $\square$

The above proposition applies to the action of $${\text {Aut}}({\mathbb {F}}_n)$$ on $${\mathbb {F}}_n=\langle x_1,\ldots ,x_n\rangle $$ for $$n\geqslant 2$$, since $$x_i^m = x_j^{-1}\cdot x_j^{} x_i^m$$$$(j\ne i)$$ has $$\ell (x_i^m )\leqslant 2$$.

### Corollary 11

If ${\text {Aut}}({\mathbb {F}}_n)$ has property (*T*), then the subgroup

$$\begin{aligned} \Gamma =\left\{ \theta \in {\text {Aut}}({\mathbb {F}}_{n+1}) :\theta ({\mathbb {F}}_n)\subseteq {\mathbb {F}}_n\right\} \end{aligned}$$

in ${\text {Aut}}({\mathbb {F}}_{n+1})$ has property (*T*).

### Proof

Let $\left\{ x_i\right\} _{i=1}^{n+1}$ denote the standard generating set of ${\mathbb {F}}_{n+1}$. Any element $\theta$ as above satisfies $\theta (x_{n+1})=a x_{n+1}^{\pm 1} b$ for some $a,b\in {\mathbb {F}}_n$: indeed, since $\theta ({\mathbb {F}}_{n}) \subseteq {\mathbb {F}}_n$, $\theta$ has a word representative which involves neither letters $L_{i,n+1}^{\pm 1}$ nor $R_{i, n+1}^{\pm 1}$ for $1\leqslant i \leqslant n$. Therefore any letter $L_{n+1, i}^{\pm 1}$ ($R_{n+1, i}^{\pm 1}$ respectively) of $\theta$ results in multiplying $x_{n+1}$ by a word from ${\mathbb {F}}_n$ on the left (right respectively). This means that $\Gamma$ is isomorphic to $({\text {Aut}}({\mathbb {F}}_n)\times {\mathbb {Z}}/2)\ltimes ({\mathbb {F}}_n\times {\mathbb {F}}_n)$, where ${\text {Aut}}({\mathbb {F}}_n)$ acts on ${\mathbb {F}}_n\times {\mathbb {F}}_n$ diagonally and ${\mathbb {Z}}/2$ acts on ${\mathbb {F}}_n\times {\mathbb {F}}_n$ by the flip. Since ${\text {Aut}}({\mathbb {F}}_n)\ltimes {\mathbb {F}}_n$ has property (*T*) by the above proposition, ${\text {Aut}}({\mathbb {F}}_n)\ltimes ({\mathbb {F}}_n\times {\mathbb {F}}_n)$ has property (*T*) as well. $\square$

### Remark 12

In [30, 4) p. 324] Popa asked for an example of an action of a property (*T*) group *G* on $L({\mathbb {F}}_n)$, the free group factor, so that the crossed product $L({\mathbb {F}}_n)\rtimes G$ has property (*T*). The examples considered above provide an answer to Popa’s question for $n=5$. Indeed, by the above arguments, taking $G={\text {Aut}}({\mathbb {F}}_{5})$ with its natural action on $L({\mathbb {F}}_5)$ satisfies all the necessary conditions. Another example is given by taking $G={\text {Out}}({\mathbb {F}}_5)$, in which case $L({\text {Aut}}({\mathbb {F}}_5))$ is the crossed product $L({\mathbb {F}}_5)\rtimes {\text {Out}}(F_5)$.

## ${\text {SL}}_6({\mathbb {Z}})$ and ${\text {SL}}_4({\mathbb {Z}}\langle X\rangle )$

We have also applied the symmetrized algorithm to certain other groups, for which the non-symmetrized approach as in [12, 18, 26], was not succesful. In the case of two linear groups, ${\text {SL}}_6({\mathbb {Z}})$ and ${\text {SL}}_4({\mathbb {Z}}\langle X\rangle )$ (generated by the set of elementary matrices *E*(4) and *E*(6), respectively), we obtained new estimates for the Kazhdan constants. For the first group we obtained a certified bound

$$\begin{aligned} \kappa ({\text {SL}}_6({\mathbb {Z}}), E(6)) \geqslant 0.3812; \end{aligned}$$

for the second group the certified bound is

$$\begin{aligned} \kappa ({\text {SL}}_4({\mathbb {Z}}\langle X\rangle ), E(4)) \geqslant 0.2327. \end{aligned}$$

## Final remarks

**Smallest radius for existence of a solution** As mentioned in the introduction, one of the reasons for our approach not producing an answer in the case of ${\text {Aut}}({\mathbb {F}}_4)$ could be that the Eq. (1) does not have a solution where all $\xi _i$’s are supported in ball of radius 2. It is in general unclear, for a group with property (*T*), what is the smallest radius *r* such that (1) has a solution on the ball of radius *r*. It is equally unclear what is the radius *r* for which the optimal $\lambda$ is attained (if it exists).

We have not been able to reprove property (*T*), using the method presented here, for ${\text {SL}}_3({\mathbb {Z}}[X])$ on the ball of radius 2. Recall that for ${\text {SL}}_3({\mathbb {Z}}[X])$ property (*T*) was proved by Shalom [33] and Vaserstein [35], as well as by Ershov and Jaikin-Zapirain [10].

Note also that the bound given in [12] on a ball of radius 2 was better for ${\text {SL}}_4({\mathbb {Z}})$ than for ${\text {SL}}_3({\mathbb {Z}})$. All this suggests that to detect property (*T*) (or estimate the Kazhdan constant) of ${\text {SL}}(n,{\mathbb {Z}}[X_1,\ldots ,X_k])$ one needs larger ball as *k* increases and smaller ball as *n* increases (although the Kazhdan constant itself gets smaller).

## Footnotes

1 Note that given two vectors of values on ${\mathbf {x}}$ which represent elements *x*, *y* of *RG*, using only the division table (i.e. without referring to the elements of ${\mathbf {x}}$) we can compute $x^*y$, but not *xy*.

2 The initial computations have been performed on a cluster in PL-Grid network.
